# A New Chalcone Derivative with Promising Antiproliferative and Anti-Invasion Activities in Glioblastoma Cells

**DOI:** 10.3390/molecules26113383

**Published:** 2021-06-03

**Authors:** Daniel Mendanha, Joana Vieira de Castro, Joana Moreira, Bruno M. Costa, Honorina Cidade, Madalena Pinto, Helena Ferreira, Nuno M. Neves

**Affiliations:** 1 3B’s Research Group, I3Bs—Research Institute on Biomaterials, Biodegradables and Biomimetics, University of Minho, Headquarters of the European Institute of Excellence on Tissue Engineering and Regenerative Medicine, AvePark, Parque de Ciência e Tecnologia, Zona Industrial da Gandra, Barco, 4805-017 Guimarães, Portugal; daniel.mendanha@i3bs.uminho.pt (D.M.); joana.castro@i3bs.uminho.pt (J.V.d.C.); 2 ICVS/3B’s-PT Government Associate Laboratory, 4805-017 Guimarães, Portugal; bfmcosta@med.uminho.pt; 3 Laboratory of Organic and Pharmaceutical Chemistry, Department of Chemical Sciences, Faculty of Pharmacy, University of Porto, Rua de Jorge Viterbo Ferreira 228, 4050-313 Porto, Portugal; up201302558@edu.ff.up.pt (J.M.); hcidade@ff.up.pt (H.C.); madalena@ff.up.pt (M.P.); 4 Interdisciplinary Centre of Marine and Environmental Research (CIIMAR), University of Porto, Edifício do Terminal de Cruzeiros do Porto de Leixões, Avenida General Norton de Matos s/n, 4450-208 Matosinhos, Portugal; 5 Life and Health Sciences Research Institute (ICVS), School of Medicine, Campus Gualtar, University of Minho, 4710-057 Braga, Portugal

**Keywords:** glioblastoma, chalcone, cell death, drug delivery, liposomes

## Abstract

Glioblastoma (GBM) is the most common and most deadly primary malignant brain tumor. Current therapies are not effective, the average survival of GBM patients after diagnosis being limited to few months. Therefore, the discovery of new treatments for this highly aggressive brain cancer is urgently needed. Chalcones are synthetic and naturally occurring compounds that have been widely investigated as anticancer agents. In this work, three chalcone derivatives were tested regarding their inhibitory activity and selectivity towards GBM cell lines (human and mouse) and a non-cancerous mouse brain cell line. The chalcone 1 showed the most potent and selective cytotoxic effects in the GBM cell lines, being further investigated regarding its ability to reduce critical hallmark features of GBM and to induce apoptosis and cell cycle arrest. This derivative showed to successfully reduce the invasion and proliferation capacity of tumor cells, both key targets for cancer treatment. Moreover, to overcome potential systemic side effects and its poor water solubility, this compound was encapsulated into liposomes. Therapeutic concentrations were incorporated retaining the potent in vitro growth inhibitory effect of the selected compound. In conclusion, our results demonstrated that this new formulation can be a promising starting point for the discovery of new and more effective drug treatments for GBM.

## 1. Introduction

Glioblastoma (GBM), the most common malignant type of primary brain tumor in the central nervous system (CNS), presents specific pathophysiology characteristics, such as nuclear atypia, increased mitotic activity, microvascular proliferation, and tissue necrosis [1,2]. The World Health Organization (WHO) classifies this type of tumor with a grade IV, the highest into its malignant scale of CNS tumors [1]. GBM has an approximate annual incidence rate of 0.59 to 5 per 100,000 people, with studies indicating a rise in incidence in the last few years [3]. The mean age of primary GBM patients is 62 years old, and the median overall survival is approximately 14.6 months, revealing the lack of efficacy of current treatments [4].

The currently available treatment consists of the maximal surgical resection of the tumor, followed by a radiotherapy treatment schedule conjugated with the use of the alkylating agent temozolomide (TMZ) [4,5,6]. The high invasiveness capacity of GBM, together with its intra- and intertumoral heterogeneity, leads to very different patient responses to the treatment and makes difficult the development of alternative and effective therapeutic regimens [7,8]. As recently reviewed, some promising drugs have been investigated in clinical trials, although with variable efficacy [9]. Consequently, a better alternative to the current treatment is yet to be established.

The development of new drug-based therapies for GBM is highly dependent on the ability of the drugs to cross the blood–brain barrier (BBB) and reach the tumor site in therapeutic concentrations. The BBB is a tight structure responsible for maintaining a homeostatic environment in the CNS [10,11]. The high transendothelial electrical resistance in conjugation with the presence of efflux pumps at the luminal side of the BBB prevents the crossing of lipophilic molecules, xenobiotics, toxic metabolites, and drugs [12,13]. The inability to efficiently cross the biological barriers and the systemic side effects are two of the main reasons for the limited availability of new chemotherapeutic agents for GBM treatment.

The use of new strategies to improve the pharmacokinetics and safety of newly developed as well as established drugs has been intensively investigated in the last decades. One of the most promising approaches includes the use of nanocarriers to protect and deliver the therapeutic agents at the tumor site. Among the different nanocarriers developed, liposomes present distinctive advantages as drug delivery devices, including increased targeting capacity, long-term efficacy, enhanced drug stability, reduced drug toxicity, and increased circulation time [14,15,16].

Natural and synthetic chalcones were intensively explored for a wide range of biological activities, such as antimicrobial [17], anti-inflammatory [18], antioxidant [19], and antitumor activity [20]. Considering their simple chemistry, ease of synthesis, and the large number of replaceable hydrogens facilitating chemical modifications, this class of compounds was intensively explored for cancer treatment [20]. Regarding their antitumor effects, several studies have shown that chalcone derivatives can present antiproliferative activity on cancer cells, and several molecular targets have already been identified and studied [20,21,22].

**Figure 1:**
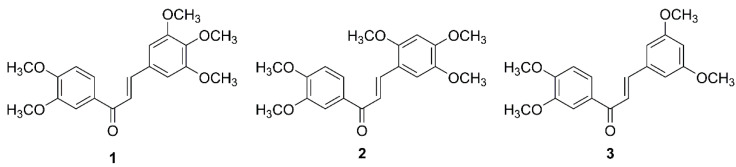
Chemical structure of chalcones **1–3**.

The ability of some types of chalcones and derivatives to induce cell death in cancer cells can bring a new class of therapeutic agents to GBM treatment. Recently, different studies using natural and synthetic chalcones against GBM have shown the potential therapeutic activity of those compounds. Different cell death mechanisms induced by these molecules have already been described, such as apoptosis mediated by caspases [21,23], autophagy [21], methuosis [24], and unfolded protein response mediated cell death [25]. We already reported several chalcones with promising growth inhibitory activity in human cancer cell lines [26,27,28,29,30,31,32]. Among these, chalcones **1**–**3** (Figure 1) revealed antiproliferative activity in A375-C5 (melanoma), MCF-7 (breast adenocarcinoma), and NCI-H460 (non-small cell lung cancer) cells [33]. Moreover, the effect of chalcones **1**–**2** in NCI-H460 demonstrated a pronounced anti-mitotic effect, blocking mitotic progression by affecting spindle assembly and, therefore, arrested cells at metaphase, culminating in cell death [33]. Considering the potential antitumor activity of these compounds in other cancer cell lines, we hypothesized that these chalcone derivatives can have a therapeutic effect on GBM cells, by inducing selective and controlled cell death. Moreover, the ability of the most promising compound to be encapsulated in liposomes while maintaining their biological effect was also accessed.

## 2. Results

### 2.1. In Vitro Cytotoxic Activity of Chalcones **1**–**3** on GBM

**Figure 2:**
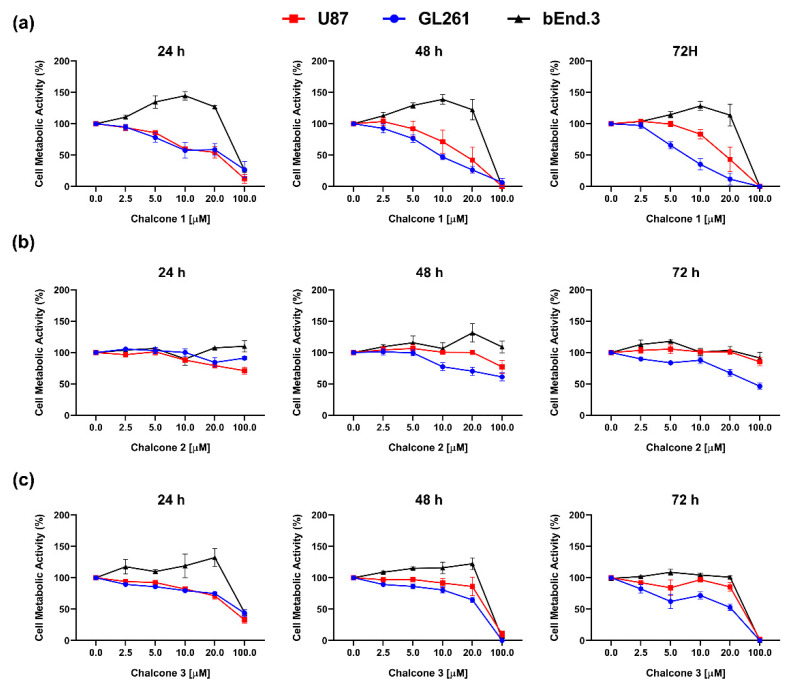
Cytotoxic effect of chalcone derivatives—Cell metabolic activity after treatment (24, 48, and 72 h) with chalcones **1** (**a**), **2** (**b**), and **3** (**c**) on GBM cell lines (U87 and GL261) and a brain endothelial cell line (bEnd.3) by MTS assay. Results are normalized to control (1% DMSO) and presented as mean ± SEM of three independent experiments.

Chalcones **1–3** were evaluated regarding their potential cytotoxic effect on GBM cells as well as on a healthy brain cell line. A range of concentrations from 2.5 µM to 100 µM of these compounds was tested to verify their effectiveness towards two different GBM cell cultures, namely human glioblastoma astrocytoma (U87) and murine glioma (GL261) cells, as well as a non-tumor cell line (bEnd.3), at different time points (Figure 2).

Compound **1** (Figure 2a) demonstrated the ability to inhibit cell metabolic activity in both GBM cell lines at different time points, which was not observed for compound **2** (Figure 2b). Regarding the chalcone derivative **3**, it was possible to observe a decrease of cell metabolic activity of GBM cell lines, with a reduction superior to 50% detected only with the maximal tested concentration of 100 µM (Figure 2c). The non-tumor endothelial cell line bEnd.3, commonly used to recreate the BBB in vitro, showed more resistance towards chalcone **1** and **3** with a decrease in metabolic activity only at 100 µM concentration. These results evidenced a potential selectivity of these compounds towards GBM cell lines. Moreover, it was possible to observe a dose-response effect on GL261 (*p* < 0.0001) and U87 (*p* < 0.0001) after chalcone **1** treatment (Figure 2a), being a time-dependent effect only observed in the GL261 cell line (*p* = 0.0003). Based on these results, the half-maximal inhibitory concentration (IC_50_) of chalcone **1** was calculated for the GL261 cell line at 24 h (25.54 µM), 48 h (10.02 µM), and 72 h (7.34 µM), and the U87 cell line at the same time points (19.50 µM, 16.51 µM, and 18.07 µM for 24, 48 and 72 h, respectively). Considering the selective and cytotoxic effect observed, the IC_50_ of chalcone **1** was used to explore its possible mechanisms of action.

### 2.2. Impact of Chalcone 1 on GBM Hallmarks

**Figure 3:**
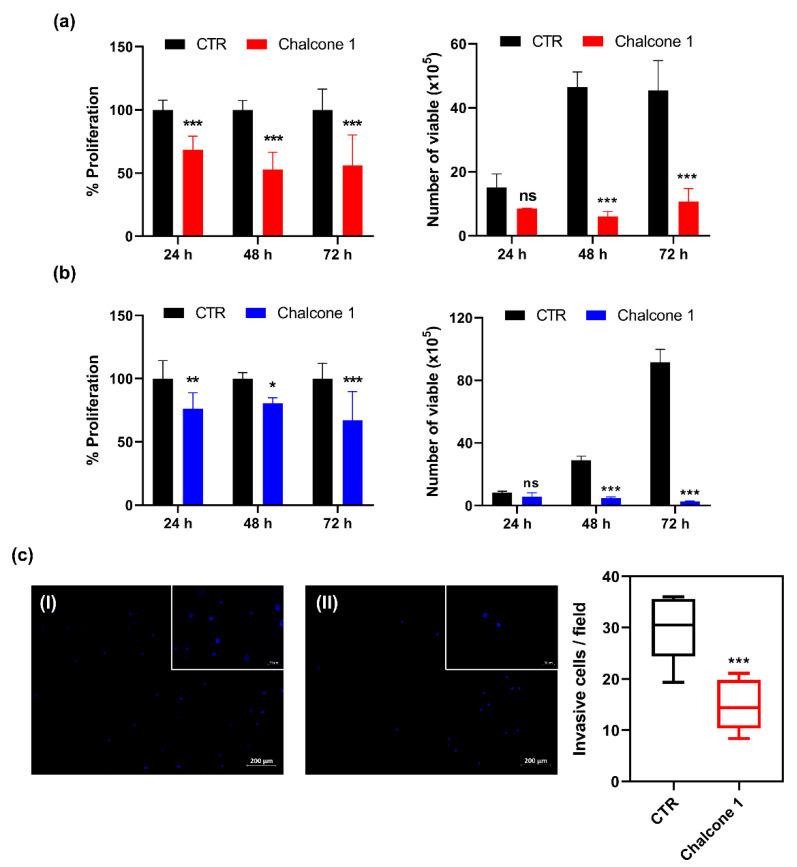
Effect of chalcone **1** on GBM hallmarks. Analyses of U87 (**a**) and GL261 (**b**) cell proliferation (left graph) and cell viability (right graph) by BrdU and Trypan Blue assay, respectively. (**c**) Analyses of U87 invasion by Matrigel invasion assay, with representative images of control (CTR; I; 0.25% DMSO) and chalcone derivative treated (II) with cell nucleus stained with DAPI. Results are presented as mean ± SD of three independent experiments. * *p* < 0.05, ** *p* < 0.01, *** *p* < 0.001 compared to CTR.

Among the wide range of pharmacologic anticancer therapies that are currently being investigated or in clinical use, the hallmark targets are the dysregulated proliferation and highly invasive profiles. The inhibitory effect of chalcone **1** on GBM cell proliferation was evaluated by assessing the BrdU incorporation during DNA synthesis. Additionally, the effect on cell viability was assessed by analyzing the membrane integrity and, consequently, the ability to exclude the dye trypan blue after treatment with the respective IC_50_ values, for 24, 48, and 72 h (Figure 3a,b). The invasive profile of U87 treated with chalcone **1** was assessed by the ability of cells to invade through a Matrigel membrane in response to chemoattractants (Figure 3c).

A statistical impairment in cell proliferation compared to non-treated cells (control) was noticeable in both GBM cell lines, after treatment with chalcone **1**, at all time points tested, with a reduction of about 40% in U87 cells and 25% in GL261 cell lines, although no time-dependent response was observable (*p* = 0.2404 for U87; *p* = 0.3277 for GL261) (Figure 3a,b: graphs on the left). Considering the total number of viable cells after treatment, it is possible to observe a significant decrease after 48 h (40 × 10^5^) and 72 h (35 × 10^5^) in U87 cells (Figure 3a: right graph). Similar results after treatment, with a reduction of 24 × 10^5^ and 88 × 10^5^ viable cells at 48 and 72 h, respectively, in GL261 cells was observed (Figure 3b: right graph). After the treatment of U87 cells (a highly invasive cell model of GBM) with chalcone **1**, a significant reduction (around 50%) of the cells’ invasion capacity was observed (*p* = 0.0005) compared to negative control cells treated with 0.25% DMSO (chalcone **1** vehicle) (Figure 3c).

### 2.3. Evaluation of Apoptosis and Cell Cycle Arrest of GBM Cells after Chalcone **1** Treatment

**Figure 4:**
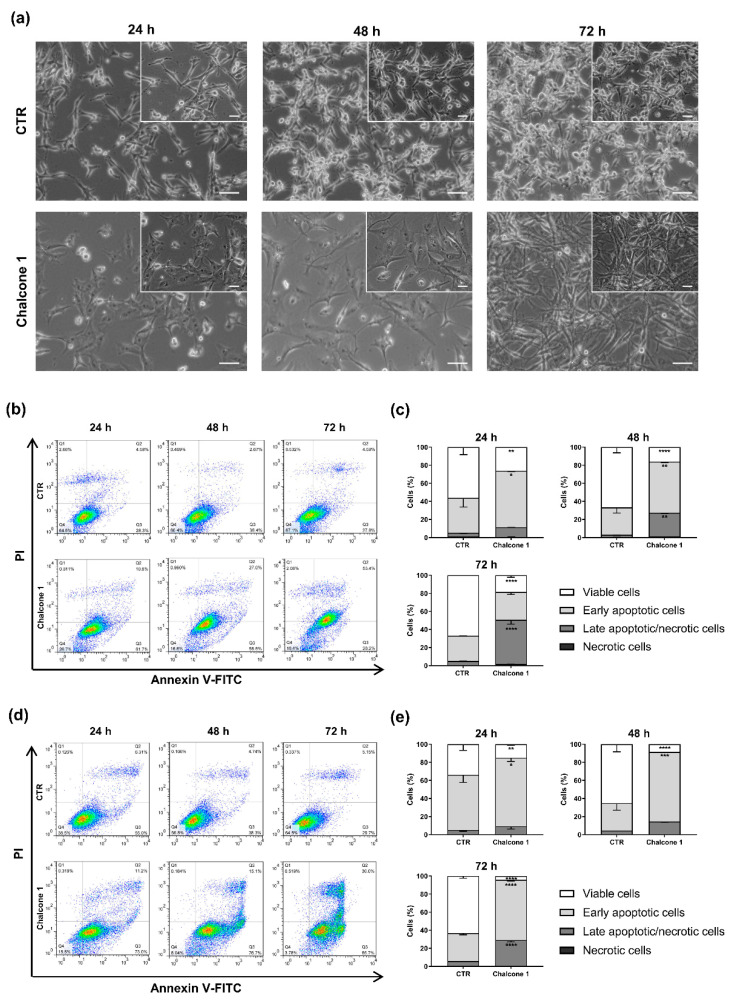
Cell death analyses after treatment with chalcone **1**. Morphological analyses of U87 cells without (control—CTR) and after treatment with chalcone **1** with the concentrations of the 24 h IC_50_ for 24, 48, and 72 h (**a**). Flow cytometry analyses of U87 (**b**) and GL261 (**d**) cell viability at different treatment times with chalcone **1** 24 h IC_50_ concentrations. Graphical representation of the percentage of U87 (**c**) and GL261 (**e**) cells in each quadrant of the dot plot. Results are presented as mean ± SD of three independent experiments. * *p* < 0.05, ** *p* < 0.01, *** *p* < 0.001, **** *p* < 0.0001 compared to CTR.

To evaluate the presence of cell death after compound **1** treatment, cells were exposed with a concentration of chalcone **1** corresponding to the 24 h IC_50_ for 24, 48, and 72 h. The Annexin V-FITC/ PI assay was used since it allows to determine if cells are viable, apoptotic, or necrotic based on the differences in plasma membrane integrity and permeability (Figure 4).

After chalcone **1** treatment, a clear difference in the morphology and density of cells was observed by phase-contrast microscopy at all time points. The U87 cells, which in 2D culture conditions have a star-like morphology, presented a different shape and a noticeable increase in size after treatment with the tested chalcone derivative (Figure 4a). The GL261 cells respond to treatment by detaching from the flask surface, an indicator of cell death (data not shown). After the chalcone **1** treatment of the U87 cells, it was possible to observe an increase in early apoptotic cells after 24 h (*p* = 0.0267) and 48 h (*p* = 0.0015) relative to the control, and an increase in late apoptotic/necrotic cells after 48 h (*p* = 0.0028) and 72 h (*p* < 0.0001). Moreover, it was noticeable the decrease in the number of viable cells at 24 (*p* = 0.0075), 48 (*p* < 0.0001), and 72 h (*p* < 0.0001; Figure 4b,c). Regarding the GL261 cell line, similar results were observed after treatment with chalcone **1**, with an increase of early apoptosis cells at 24 (*p* = 0.03008), 48 (*p* = 0.0002), and 72 h (*p* < 0.0001). After 72 h of treatment, an increase of around 20% of late apoptotic/necrotic cells was also detected (*p* < 0.0001) compared to the control condition. The number of viable cells was significantly lower at 24 (*p* = 0.0085), 48 (*p* < 0.0001), and 72 h (*p* < 0.0001) when compared to control cells (Figure 4d,e).

**Figure 5:**
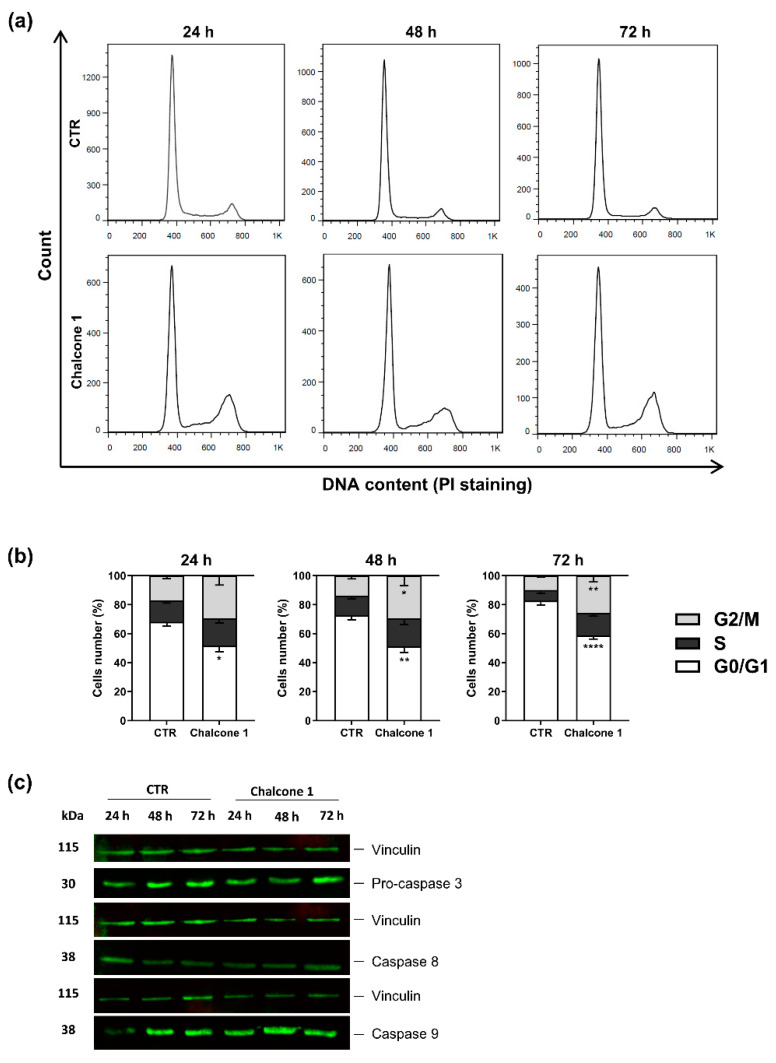
Cell death mechanisms induced by chalcone **1**. (**a**) Cell cycle profile of U87 cells treated or not (control—CTR) for 24, 48, and 72 h with chalcone **1** 24 h IC_50_ concentration. (**b**) Quantification of cells in different phases of the cell cycle after treatment. (**c**) Representative immunoblot of U87 cells treated with chalcone **1**. Results are presented as mean ± SD of three independent experiments. * *p* < 0.05, ** *p* < 0.01, **** *p* < 0.0001 compared to CTR.

Considering the percentage of late apoptotic/necrotic cells in the U87 cell line after treatment, we explored the possible mechanisms that could be responsible for this process. The U87 cells were treated with chalcone **1** at a concentration of the 24 h chalcone **1** IC_50_ and the cell cycle was analyzed by propidium iodide (PI) staining using flow cytometry, while the expression of caspases was analyzed by the western blot assay (Figure 5).

The DNA content, correlated with the brightness of the stoichiometric dye (PI) detected in each cell, was quantified by flow cytometry after treatment. The increase in PI staining (Figure 5a,b) showed an arrest of chalcone **1** treated cells in the G_2_/M checkpoint after 48 (*p* = 0.0362) and 72 h (*p* = 0.0013). The expression of caspases 3, 8, and 9 were analyzed to access possible apoptotic pathways triggered by chalcone **1** treatment. No significant differences in the expression of early precursor caspases 8 and 9 were detected. Moreover, only pro-caspase 3 expression was observed in the treated and non-treated cells, with no cleavage and, consequently, no caspase 3 expression being detected in the conditions (Figure 5c).

### 2.4. Characterization and Biological Activity of Liposomes Incorporating Chalcone **1**

**Figure 6:**
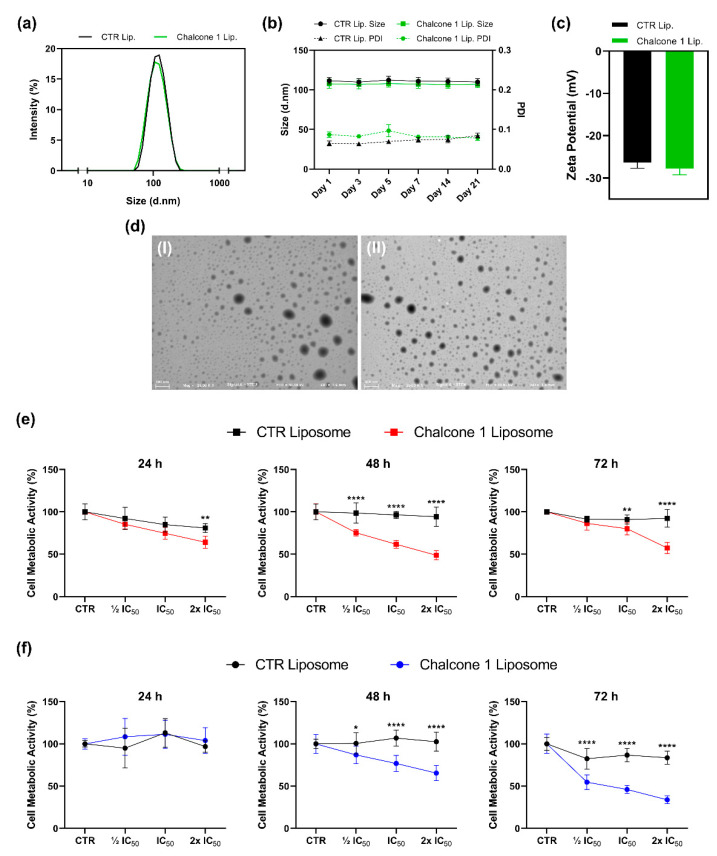
Characterization and biological activity of liposomes incorporating chalcone **1**. Analyses of the size distribution (**a**), stability (**b**), and zeta potential (**c**) of liposomes. Morphological analyses of empty liposomes (I) and liposomes incorporating chalcone **1** (II) by STEM (**d**). U87 (**e**) and GL261 (**f**) GBM cells metabolic activity was quantified by MTS assay in the absence (control—CTR) and presence of liposomes incorporating chalcone **1** at concentrations of ½ IC50, IC50, and 2x IC50 at the different time points. Results are presented as mean ± SD of three independent experiments. * *p* < 0.05, ** *p* < 0.01, **** *p* < 0.0001 compared to CTR.

Considering that one of the main limitations of chalcones is their poor water solubility, the encapsulation of chalcone **1** in a nanocarrier was performed and the biological activity of the resulting liposomal formulation was accessed (Figure 6).

Regarding the encapsulation efficiency, we were able to load 49.60 ± 6.29% of chalcone **1** into liposomes. The size of liposomes incorporating (107.6 ± 5.4 nm) or not incorporating (111.6 ± 3.8 nm) chalcone **1** did not present significant differences (*p* = 0.1095; Figure 6a). Moreover, the two suspensions presented a homogeneous size (polydispersity index—PDI inferior to 0.1) and a high degree of stability (no significant variation of size) for 21 days (Figure 6b). Similar (*p* = 0.0618) and significant negative charges were also presented by both liposomes without (−26.39 ± 1.22 mV) and incorporating chalcone **1** (−27.75 ± 1.38 mV; Figure 6c). Moreover, liposomes with this chalcone derivative maintained a spherical morphology (Figure 6d).

Different concentrations of chalcone **1** encapsulated into liposomes, based on the IC_50_ previously calculated, were added to the two GBM cell cultures to evaluate if its cytotoxic potential is maintained. For this evaluation, the cell metabolic activity was analyzed by MTS assay. After treatment, the U87 cells’ metabolic activity was significantly reduced in a dose-response manner at the different time points tested (*p* < 0.0001). The empty liposomes showed reduced cytotoxicity towards GBM cells. Indeed, despite a maximal of 20% inhibition after 24 h was observed, cells were able to recover their normal metabolic activity after 48 and 72 h of culture (Figure 6e). Similar results were observed with the GL261 cell line, being a dose-dependent response observed at 48 (*p* = 0.0002) and 72 h (*p* < 0.0001), although no differences were found at 24 h (*p* = 0.0877; Figure 6f).

## 3. Discussion

Despite the resources and the aggressive treatment regimen, GBM patients still present a very poor prognosis. Between the wide range of pharmacologic anticancer therapies that are currently being investigated or in use in the clinic, the selectivity and the ability to induce controlled cell death of carcinogenic cells are crucial steps in the development of new, safe, and effective pharmacological strategies. From the three chalcone derivatives tested, chalcone **1** showed the most promising effects (Figure 2). Indeed, considering the selectivity observed, chalcone **1** presented higher cytotoxicity in GBM cells comparing to brain endothelial cells, at the same concentrations of the other two derivatives. Moreover, considering the importance of a thorough assessment of the compounds in different cells that can model the heterogeneity found in this type of tumor, we observed different time-dependent responses between the two GBM cell lines considered. Importantly, the IC_50_ values calculated for chalcone **1** are in the range proposed by different studies for this class of compounds against GBM [25,34] and other cancer cells [33,35]. The identification of new candidate treatments for cancer, and particularly for GBM, are closely associated with the ability to inhibit one or more of the cancer hallmarks [36,37]. Therefore, we evaluated the ability of chalcone **1** to inhibit GBM cell proliferation and invasion (Figure 3). Indeed, this chalcone derivative was effective in decreasing the deregulated proliferation, and the high invasion capacity of GBM cells was compromised when treated with chalcone **1** (Figure 3), showing its potential to inhibit distinct mechanisms of cancer progression. Although several studies showed the ability of different chalcones and their derivatives to inhibit the proliferation of glioma cells [34,38], studies covering its impact on invasion are more limited. However, as described by us (Figure 3c), other studies reveal the potential of chalcone impact on cancer cell invasion. For instance, the regulation of the tropomyosin 1 gene was suggested as a possible mechanism for decreasing the invasion of glioma cells when exposed to chalcone derivatives [39], but more studies are needed to establish this link. The use of chalcones for the treatment of different cancers has also shown that they can reduce cell migration and invasion by reducing MMP-2 and MMP-9 activity through the downregulation of JNK signaling pathways [40]. Finally, the modulation of NF-κB and Akt signaling pathways by a chalcone derivative was also proposed as a mechanism of action for the decrease of colon cancer cell invasion and migration [41].

The evasion of programmed cell death is a key hallmark of cancer that contributes to tumor formation and progression [36]. The blockage of cell death in cancer cells is closely associated with chemotherapeutic resistance, and the inability to maintain an intact signal transduction pathway leading to cell death [42]. Therefore, the ability of chalcone **1** to induce GBM cell death was evaluated, demonstrating its effectiveness in inducing GBM cell death (Figure 4). Moreover, the detection of a large percentage of apoptotic cells instead of necrotic cells after treatment with chalcone **1**, suggests that this chalcone derivative is promoting a programmed cell death via apoptotic pathways. We observed an increase of late apoptotic cells over time which demonstrates a continuous response to treatment. Although different cell death mechanisms have been previously reported in GBM in response to chalcone derivatives [24,25], apoptosis is the most commonly reported [21,23], as observed in this study (Figure 4). We demonstrated that the treatment of GBM cells with chalcone **1** led to visible cell morphological alterations (Figure 4a). Indeed, it is possible to detect an increase in cell debris and morphological changes in the membrane that are visual indications of cells under apoptosis, as previously reported [43]. The current chemotherapeutic agent used in the clinic, TMZ, presents a mechanism of action by adding methyl groups at N^7^ and O^6^ sites on guanines and O^3^ position on adenines leading to deficient DNA replication, a G_2_/M checkpoint arrest, and, consequently, cell death [44,45]. We also observed an arrest of cells in G_2_/M after treatment (Figure 5a,b), explaining the decrease of proliferation on chalcone **1** treated cells and showing a possible mechanism responsible for the activation of apoptotic pathways. Indeed, the arrest in this checkpoint was already reported for different chalcone derivatives in GBM treatment, being a commonly reported feature when developing pharmacological anticancer drugs [46].

During apoptosis, two different molecular pathways, extrinsic and intrinsic, can be activated to induce cell death. In the extrinsic pathway, proapoptotic ligands activate cell surface receptors associated with cell death that lead to the formation of a death-signaling inducing complex. The intrinsic pathway is activated inside the cell in response to cell stress, such as DNA damage or oxidative stress [42]. In both pathways exist a proteolytic activation of caspases leading to cell changes, such as chromatin condensation, DNA fragmentation, or membrane blebbing [42]. Therefore, in this work, after chalcone **1** treatment the expression of caspases 9 and 8, associated with intrinsic and extrinsic pathways, respectively, and the expression of the effector caspase 3 were analyzed. Interestingly, we did not observe differences in the caspase expression after treatment, observing similar levels of expression of caspases 8 and 9 in both the control and treated cells (Figure 5c). The results can be explained by deregulated apoptosis pathways associated with GBM [42]. The absence of expression of caspase 3, can suggest that apoptosis is executed by other effector caspases, such as caspases 6 or 7, or by caspase-independent cell death [47]. However, more studies are needed to clarify the apoptotic pathways activated after treatment with chalcone derivatives.

The poor water solubility, the reticulum endoplasmic system, and the inability to cross the BBB are only some of the reasons that justify the increased emphasis on the development of drug delivery systems for GBM treatment. Particularly, liposomes have been used to increase the efficacy of chemotherapeutic agents in GBM for over a decade [48]. Therefore, in this work, a liposomal system able to carry chalcone **1** was developed, using commonly available phospholipids (Figure 6). This liposome suspension presented long-term stability with a homogeneous size (PDI < 0.2) around 100 nm. Consequently, they present key characteristics of nanocarriers used to target GBM (Figure 6a,b) [49,50]. Indeed, drug delivery devices should present a size <150 nm to enter and exit fenestrated capillaries in the tumor microenvironment and reach the carcinogenic cells [51]. After incorporation in liposomes, chalcone **1** was able to maintain the cytotoxic effect. However, the results suggest that the liposomal formulation can take more time to present similar cytotoxic effects to free chalcone, which can be related to liposome internalization. Indeed, we observed an increased cytotoxic effect after 48 and 72 h (Figure 6e,f).

**Figure 7:**
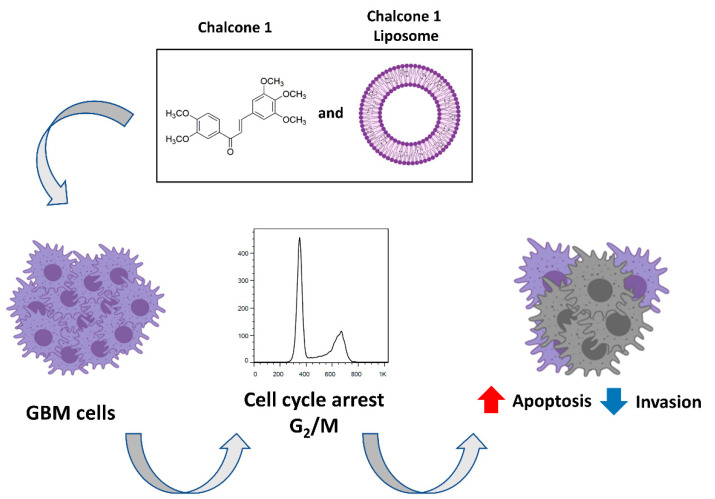
Impact and mechanism of action of chalcone **1** in GBM cell lines.

Gathering all the results, the proposed mechanisms of action for chalcone **1** are presented in Figure 7.

## 4. Materials and Methods

### 4.1. Reagents

Dulbecco’s Modified Eagle Medium (DMEM), fetal bovine serum (FBS), and trypLE Express were purchased from Life Technologies (Carlsbad, CA, USA). CellTiter 96^®^ AQueous One Solution was purchased from Promega (Madison, WI, USA). ELISA BrdU (colorimetric) was acquired from Roche (Basel, Switzerland). BD BioCoat^TM^ Matrigel^TM^ Invasion Chambers were acquired from Corning^®^ (Corning, New York, NY, USA). The dead cell apoptosis kit with Annexin V-FITC/ PI was obtained from BD Bioscience (Franklin Lakes, NJ, USA). RNAse A and PI were purchased from Invitrogen (Carlsbad, CA, USA). Anti-caspase 8 and anti-caspase 9 antibodies were purchased from Santa Cruz Biotechnology (Dallas, TX, USA). IRDye 800CW Goat anti-mouse IgG and IRDye 800CW Goat anti-rabbit IgG were acquired from LI-COR (Lincoln, NE, USA). Anti-caspase 3 was purchased from Abcam (Cambridge, UK). L-α-phosphatidylcholine (PC) and anti-vinculin antibody were obtained from Sigma-Aldrich (St. Louis, MI, USA). 1,2-Dioleoyl-sn-glycerol-3-phosphoserine sodium salt (Lipoid PS 18:1/18:1) was obtained from Lipoid GmbH (Ludwigshafen, Germany) and LabAssay Phospholipid was obtained from Wako (Osaka, Japan).

### 4.2. Chalcone Derivatives

Details concerning the synthesis of chalcones **1**–**3** (Figure 1) were previously reported [33]. Briefly, to a solution of 3,4-dimethoxyacetophenone (1.01–7.93 mmol) in methanol (25 mL), an aqueous solution of 40% sodium hydroxide (methanol/water) was added until pH 13–14, under stirring and on ice. Then, a solution of appropriately substituted benzaldehyde (2.03–15.86 mmol) in methanol was slowly added to the reaction mixture. The reaction was submitted to 2 h of microwave irradiation of 180 W of power, with the final temperature of 69 °C. Upon completion, the reaction mixture was poured into ice and the pH was adjusted to approximately 7 with diluted hydrochloride acid. For chalcone **1**, the resulting residue was filtered, washed with water, and purified by crystallization with methanol (yield: 81%). For chalcones **2** and **3**, the resulting residue was taken in chloroform and further rinsed with water, dried over anhydrous sodium sulfate, evaporated under reduced pressure, and the obtained residue was purified by flash column chromatography (n-hexane: ethyl acetate, 6:4; yield: 67 and 62%, respectively). The structure elucidation of compounds was established by ^1^H and ^13^C NMR techniques and data were in accordance with the data previously reported [52,53]. Compounds **1**–**3** were dissolved in DMSO at a stock concentration of 10 mM and kept at −20 °C. Dilutions were freshly prepared to achieve the desired concentrations before each experiment.

### 4.3. Cell Lines and Culture Conditions

Two different established GBM cell line models, U87 (human) and GL261 (murine), and a brain endothelial cell line, bEnd.3 (mouse) were used during the experiments. The cell lines were maintained in DMEM supplemented with 10% FBS and 1% penicillin-streptomycin and incubated at 37 °C in a humidified 5% (*v/v*) CO_2_ atmosphere.

### 4.4. Cell Metabolic Activity

The two GBM cell lines (U87 and GL261) and the brain endothelial cell line (bEnd.3) were seeded in 96-well plates at 3000 cells/100 µL (GL261 and bEnd.3) and 2000 cells/100 µL (U87) per well. The cells were allowed to adhere for 24 h. Then, they were exposed to different concentrations of chalcones **1**, **2**, and **3** for 24, 48, and 72 h. The control was performed with 1.0% DMSO (compound vehicle), matching the maximum amount of this organic solvent used with the tested compounds. The cytotoxicity and the IC_50_ of chalcone derivatives were assessed using the MTS assay (CellTiter 96^®^ AQueous One Solution). Briefly, DMEM without FBS and phenol red was mixed with MTS reagent (5:1 volume ratio) and added to each condition. Cells were incubated for 3 h at culture conditions and then the absorbance was read in triplicate at 490 nm using a microplate reader (Synergy HT, Bio-Tek, Winooski, USA). GraphPad Prism 8 software was used to calculate the IC_50_ values from three independent experiments, each one in triplicate, applying sigmoidal dose-response (variable slope) non-linear regression after logarithmic transformation.

The same experimental conditions were used to evaluate the cytotoxicity of liposomes with chalcone **1** towards the GBM cell lines. Empty liposomes at the same lipidic concentration necessary to carry the tested chalcone **1** concentrations were used as controls. Results were normalized to the condition cultured without liposomes (only with medium).

### 4.5. Cell Viability

The U87 and GL261 GBM cells were plated at an initial density of 1.5 × 10^5^/2 mL per well in 6-well plates and grown in culture conditions. Cells were treated with chalcone **1** at the respective 24 h IC_50_ values for 24, 48, and 72 h of culture. At each time point, cells were washed, trypsinized, and the number of viable cells was counted using Countess II (Invitrogen, Carlsbad, USA) according to the manufacturer’s protocol.

### 4.6. Cell Proliferation

The U87 and GL261 cells were plated in 96-well plates at a density of 2000 cells/100 µL and 3000 cells/100 µL, respectively. Then, they were incubated for 24 h in culture conditions to adhere and grown. After this period, cells were treated with the respective 24 h IC_50_ of each cell line or 0.25% DMSO (control) for 24, 48, and 72 h. After incubation, a cell proliferation ELISA BrdU (colorimetric) was performed according to the manufacturer instructions (Roche, Basel, Switzerland). Briefly, cells were labeled by addition of 10 µL/well of BrdU (final concentration of 10 µM) and incubated for two additional hours in culture conditions, allowing for its incorporation into the DNA. After labeling, the medium was removed by suction and cells were fixed with FixDenat solution for 30 min. The solution was removed, and the Anti-BrdU-POD solution was incubated for 90 min at room temperature. The antibody conjugate was removed, and the samples were washed three times with washing solution. The samples were incubated with substrate solution for 30 min at room temperature, and the absorbance of the samples was subsequently read at 370 nm (reference wavelength of 492 nm) in a microplate reader (Synergy HT, Bio-TEK, Winooski, USA). The results presented represent three independent experiments (in triplicate), after removing blank values.

### 4.7. Cell Invasion

The U87 cell invasion was evaluated using BD BioCoat^TM^ Matrigel^TM^ Invasion Chambers (Corning^®^, Corning, USA). Briefly, inserts with Matrigel were rehydrated for two hours at cell culture conditions, using DMEM without FBS. The U87 cells were seeded at 2.5 × 10^4^ cells at the top of each insert, in DMEM supplemented with 1% FBS. DMEM supplemented with 10% FBS was used in the lower chamber to work as a chemoattractant to the cells. Cells were incubated for 24 h in cell culture conditions with a concentration of chalcone **1** corresponding to the 24 h IC_50_ or 0.25% DMSO (control). After incubation, the non-invasive cells were removed from the upper part of the membrane by gently swabbing twice. The invasive cells (placed in the bottom part of the membrane) were fixed with cold methanol for 2 min and washed two times with PBS. The inserts were left to air dry, and the membrane was stained with DAPI for 5 min. The membranes were cut from the inserts and analyzed using an inverted fluorescent microscope (Zeiss, Oberkochen, Germany) with a coupled camera (Axio Imager Z1m, Zeiss, Oberkochen, Germany). Eight photos per sample covering a broad area of the membrane at 5x magnification were taken for nucleus counting. Results are presented as the mean number of cells counted per field, corresponding to three independent experiments in triplicates.

### 4.8. Cell Cycle Analyses

The U87 cells were plated at an initial density of 1.5 × 10^5^/2 mL per well in 6-well plates and grown overnight at culture conditions. Cells were treated with 24 h chalcone **1** respective IC_50_ for 24, 48, and 72 h. Cells were collected, pelleted, washed, and fixed in cold ethanol (70%, *v/v*) for one hour at 4 °C. After that, cells were washed twice with PBS containing 0.1% triton and after centrifugation and elimination of the supernatant, 50 μL of RNAse A (100 μg/mL) was added to the pellet and incubated at room temperature for 15 min. After the incubation period, 200 μL of PI (50 μg/mL) was added, followed by flow cytometry analyses. A total of 20,000 events were acquired by a flow cytometer (BD FACSCalibur, BD Biosciences, New Jersey, USA). The percentage of cells in each phase was analyzed by FlowJo software (version 7.6). At least three independent biological replicates were performed.

### 4.9. Cell Death

Cell death was evaluated after treatment with chalcone **1** by Annexin V-FITC/ PI, according to the manufacturer’s instructions (BD Biosciences, New Jersey, USA). Briefly, the U87 and GL261 GBM cells were plated at an initial density of 1.5 × 10^5^/2 mL per well in 6-well plates and grown overnight at culture conditions. Cells were treated for 24 h with chalcone **1** at the respective IC_50_ for 24, 48, and 72 h. Floating and adherent cells were collected and centrifugated (300× *g*, 5 min). After supernatant elimination, the pellet was resuspended in 100 µL of 1x annexin-binding buffer and, then, 5 μL of FITC annexin V and 1 μL of PI were added. Samples were incubated for 15 min at room temperature in the dark. After the incubation period, 400 µL of 1x annexin-binding buffer was added to each sample, followed by flow cytometry analysis. A total of 20,000 events were acquired by a flow cytometer. The percentage of cells in each phase was analyzed using the FlowJo 7.6 software.

### 4.10. Western Blot Analyses

U87 cells were seeded in 6 well plates at a cell density of 1 × 10^5^ per well and left to adhere for 24 h. Cells were then treated with chalcone **1** at a concentration corresponding to the 24 h IC_50_, for 24, 48, and 72 h. After treatment, cells were washed with PBS and 200 µL of RIPA buffer (Sigma-Aldrich, St.Louis, USA) was added to each well. Cells were scraped and collected from the wells and incubated in ice for 30 min while being vortexed several times during this period. Lysates were centrifuged at 18,000× *g* for 16 min at 4 °C and the supernatant was collected for protein quantification using the Pierce^TM^ BCA Protein Assay Kit (Thermo Scientific, Waltham, USA). Fifteen µg of total protein of each sample were separated in a 12.5% polyacrylamide gel (120 V) and transferred to a nitrocellulose membrane using a semi-dry protocol. After protein transference, membranes were blocked one hour with 5% BSA in 1x TBS-T and incubated overnight at 4 °C with primary antibodies of anti-caspase 3 (1:5000), anti-caspase 8 (1:200), anti-caspase 9 (1:200), and anti-vinculin (1:200) prepared in the blocking solution. Then, the membranes were washed three times with TBS-T, and the secondary antibodies, namely IRDye 800CW Goat anti-mouse IgG (1:15,000) and IRDye 800CW Goat anti-rabbit IgG (1:15,000), were added to the membrane for one hour at room temperature in the dark. After incubation, the membranes were washed three times for 5 min with TBS-T, and the protein bands fluorescence analyzed using the Odyssey Fc imaging system (LI-COR, Lincoln, USA).

### 4.11. Liposomes Production and Characterization

#### 4.11.1. Liposomes Preparation

Liposomes were prepared by the thin-film hydration method followed by extrusion [54]. Briefly, a lipid film of phosphatidylcholine/phosphatidylserine at a ratio of 7:3 *(n/n)* was obtained after ethanol and dichloromethane complete evaporation in a rotatory evaporator. Then, the film was hydrated with PBS (pH 7.4) and vigorously vortexed to obtain multilaminar vesicles (MLVs). The MLVs were subsequently extruded 23 times through polycarbonate filters of 0.1 µm pore diameter, using an Avanti-Mini Extruder (London, UK). To obtain liposomes incorporating chalcone **1**, this compound was solubilized in a solution of ethanol/chloroform (3:1, *v/v*) and mixed with the lipid solution at 1:25 *(n/n).* To remove the not encapsulated chalcone, PD-10 desalting columns containing Sephadex G-25 were used according to the manufactures’ instructions (GE Healthcare, Chicago, USA).

The determination of phosphatidylcholine concentration in the liposome samples was performed using an enzymatic method, namely LabAssay Phospolipid (FUJIFILM Wako, Japan). Briefly, samples were incubated for 5 min at 37 °C with Chromogen Substrate, and the absorbance acquired at 600 nm using a microplate reader.

#### 4.11.2. Encapsulation Efficiency Determination

The determination of chalcone **1** encapsulated in the liposomes was performed by the total disruption of the liposomes with ethanol followed by measuring the absorbance of the samples at 347 nm using a microplate reader. The encapsulation efficiency (*EE%*) was determined according to the following equation:

*EE%* = (*Wt*/*Wi*) ∗ 100

where *Wt* is the weight of chalcone **1** present in the liposomes after non-encapsulated compound separation and *Wi* corresponds to the initial weight of chalcone **1** added to the liposome formulation.

#### 4.11.3. Size Distribution and Zeta Potential of the Liposomes

The size, PDI, and zeta potential of liposome suspensions (500 µM) were analyzed by Dynamic Light Scattering using disposable cuvettes and laser Doppler micro-electrophoresis using a dip cell, at 25 ± 0.1 °C, in a Malvern Zetasizer NS (Malvern Instruments, Malvern, UK) equipment. For storage stability assessment, liposomes were kept at 4 °C under static conditions for 21 days, and at defined time points the previous analyses were performed.

#### 4.11.4. STEM Analyses

Scanning-transmission electron microscopy (STEM) was used to assess the morphology of the liposomes. The liposome samples were diluted at a final lipidic concentration of 20 µM in HEPES solution to avoid the presence of PBS salts after samples drying. The samples were placed in carbon film grids, left to dry overnight, and analyzed by High-Resolution Field Emission Scanning Electron Microscopy (Auriga Compact, ZEISS, Oberkochen, Germany).

### 4.12. Statistical Analyses

GraphPad Prism 8 software (GraphPad Software Inc., San Diego, USA) was used to perform statistical analyses. Parametric tests were applied, and an independent t-test was used when comparing two groups. To compare two or more groups at different time points and with different conditions, a two-way ANOVA was applied, followed by Sidak´s multiple comparison tests. Results are presented as normalized mean ± SD or mean ± SEM, and statistical significance was defined as *p* < 0.05 for a 95% confidence interval.

## 5. Conclusions

In this work, we report evidence suggesting a potential new chalcone derivative (**1**) with cytotoxicity as well as antiproliferative and anti-invasion activities towards GBM cells. Moreover, at therapeutic concentrations, chalcone **1** presented lower cytotoxicity towards the brain endothelial cell line. The induction of apoptosis by the selected chalcone derivative in GBM cells was triggered by the cell cycle arrest in the G_2_/M checkpoint. Chalcone **1** was successfully encapsulated in liposomes while maintaining its therapeutic activity. Therefore, the development of liposomes incorporating chalcone derivatives has the potential to provide a new treatment alternative for GBM.

## Data Availability

Not applicable.

## Sample Availability

Samples of the compounds are available from the corresponding author.
